# Immune-Related Functional Differential Gene Expression in Koi Carp (*Cyprinus carpio*) after Challenge with *Aeromonas sobria*

**DOI:** 10.3390/ijms19072107

**Published:** 2018-07-20

**Authors:** Omkar Byadgi, Yao-Chung Chen, Shun Maekawa, Pei-Chyi Wang, Shih-Chu Chen

**Affiliations:** 1 Department of Veterinary Medicine, College of Veterinary Medicine, National Pingtung University of Science and Technology, Pingtung 91201, Taiwan; omkarcof1@gmail.com (O.B.); r02632007@gmail.com (Y.-C.C.); shun84topaz04@gmail.com (S.M.); 2 International Degree Program of Ornamental Fish Science and Technology, International College, National Pingtung University of Science and Technology, No. 1, Shuefu Road, Neipu, Pingtung 91201, Taiwan; 3 Southern Taiwan Fish Disease Center, National Pingtung University of Science and Technology, No. 1, Shuefu Road, Neipu, Pingtung 91201, Taiwan; 4 Research Center for Animal Biologics, National Pingtung University of Science and Technology, Pingtung, No. 1, Shuefu Road, Neipu, Pingtung 91201, Taiwan

**Keywords:** Illumina paired-end sequencing, immune response, koi carp (*Cyprinus carpio*), *Aeromonas sobria*, transcriptome

## Abstract

In order to understand the molecular basis underlying the host immune response of koi carp (*Cyprinus carpio*), Illumina HiSeq^TM^ 2000 is used to analyze the muscle and spleen transcriptome of koi carp infected with *Aeromonas sobria* (*A. sobria*). *De novo* assembly of paired-end reads yielded 69,480 unigenes, of which the total length, average length, N50, and GC content are 70,120,028 bp, 1037 bp, 1793 bp, and 45.77%, respectively. Annotation is performed by comparison against various databases, yielding 42,229 (non-redundant protein sequence (NR): 60.78%), 59,255 (non-redundant nucleotide (NT): 85.28%), 35,900 (Swiss-Prot: 51.67%), 11,772 (clusters of orthologous groups (COG): 16.94%), 33,057 (Kyoto Encyclopedia of Genes and Genomes (KEGG): 47.58%), 18,764 (Gene Ontology (GO): 27.01%), and 32,085 (Interpro: 46.18%) unigenes. Comparative analysis of the expression profiles between bacterial challenge fish and control fish identifies 7749 differentially expressed genes (DEGs) from the muscle and 7846 DEGs from the spleen. These DEGs are further categorized with KEGG. Enrichment analysis of the DEGs and unigenes reveals major immune-related functions, including up-regulation of genes related with Toll-like receptor signaling, complement and coagulation cascades, and antigen processing and presentation. The results from RNA-Seq data are also validated and confirmed the consistency of the expression levels of seven immune-related genes after 24 h post infection with qPCR. Microsatellites (11,534), including di-to hexa nucleotide repeat motifs, are also identified. Altogether, this work provides valuable insights into the underlying immune mechanisms elicited during bacterial infection in koi carp that may aid in the future development of disease control measures in protection against *A. sobria*.

## 1. Introduction

*Aeromonas* (Aeromonadaceae) species are ubiquitous and can cause infections not only in humans but also in fish. *Aeromonas* are isolated from various sources, such as fresh, estuarine, or surface waters, sewage, food products, healthy or diseased fish, or animal and human feces and are ubiquitous in aquatic ecosystems [1,2,3,4,5]. *Aeromonas* diseases in fish are mainly caused by the release of two important virulence factors, namely extracellular hemolysin and aerolysin [6,7,8]. At ambient temperature, *Aeromonas* species are known as active spoilers of fish and meat [9,10]. They are opportunistic pathogens of fish and can cause outbreak during stress conditions, such as poor water quality, overcrowding, and rough handling [1,11,12].

*Aeromonas* spp. are gram-negative, straight, nonspore-forming rods, generally cytochrome oxidase positive, facultatively anaerobic, and chemoorganotrophic and are characterized by their ability to grow in 0% NaCl but not in 6% NaCl [1]. *Aeromonas septicemia* causes fatal infectious disease in cold-blooded animals [13]; in humans [14,15,16], disease is often caused by the motile *Aeromonas*, particularly *Aeromonas hydrophila*, *Aeromonas sobria* (*A. sobria*), and *Aeromonas caviae*.

In this study, we concentrated on *A. sobria*, which causes septicaemia in cultured marine and freshwater fish in Taiwan. *A. sobria* infections frequently result in considerable economic loss to fish farmers in Taiwan. There are several studies of transcriptome profile in fish upon exposure to different pathogens, such as *Aeromonas hydrophila* infection in zebrafish (*Danio rerio*), grass carp (*Ctenopharyngodon idella*), darkbarbel cat fish (*Pelteobagrus vachellii*) [17,18,19] and *Vibrio anguillarum* infection in soles (*Cynoglossus semilaevis*) [20], indicating the activation of specific immune pathways after bacterial infection. Other examples include orange-spotted grouper (*Epinephelus coioides*) [21], the blunt snout bream (*Megalobrama amblycephala*) [22], the Chilean abalone *Concholepas* (Gastropoda, Muricidae) [23], grass carp (*Ctenopharyngodon idella*) [24], blowfish or fugu (*Takifugu rubripes*) [25], large yellow croaker (*Larimichthys crocea*) [26], and Nile tilapia (*Oreochromis niloticus*) [27,28], demonstrating that the pathways and immune gene expression are dependent on individual host and pathogen. In addition, other studies reported on response to immune stimuli, pathogenic infection, or environmental stress [29,30,31]. However, to the best of our knowledge, there is no information available on the differential gene expression profile for the entire fish transcriptome in response to *A. sobria* challenge and infection in koi carp.

## 2. Results

### 2.1. Transcriptome Sequence Assembly and Functional Annotation

Of 69,480 unigenes, 60,593 (87.21%) were annotated using at least one database, including 42,229 (NR: 60.78%), 59,255 (NT: 85.28%), 35,900 (Swiss-Prot: 51.67%), 11,772 (COG: 16.94%), 33,057 (KEGG: 47.58%), 18,764 (GO: 27.01%), and 32,085 (Interpro: 46.18%) unigenes. (Supplementary Materials, Table S1a,b). In total, 26,870 (63.08%) COG-annotated putative proteins were classified into 25 categories (Supplementary Materials, Figure S1). The largest functional cluster was determined to be ‘replication recombination and modification’ (2486), followed by ‘transcription’ (2359) and ‘translation, ribosomal structure, and biogenesis’ (1949).

### 2.2. Number of Differentially Expressed Genes after Aeromonas Sobria Challenge

**Figure 1:**
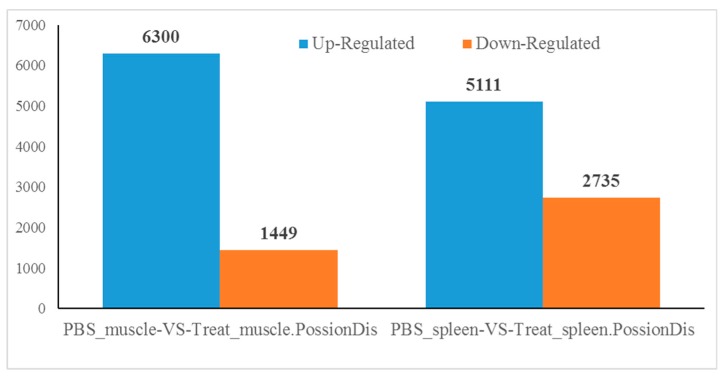
Up-regulation and down-regulation of differential expressed genes (DEGs) in muscle and spleen after challenge with *Aeromonas sobria* (*A. sobria*) in koi carp.

**Figure 2:**
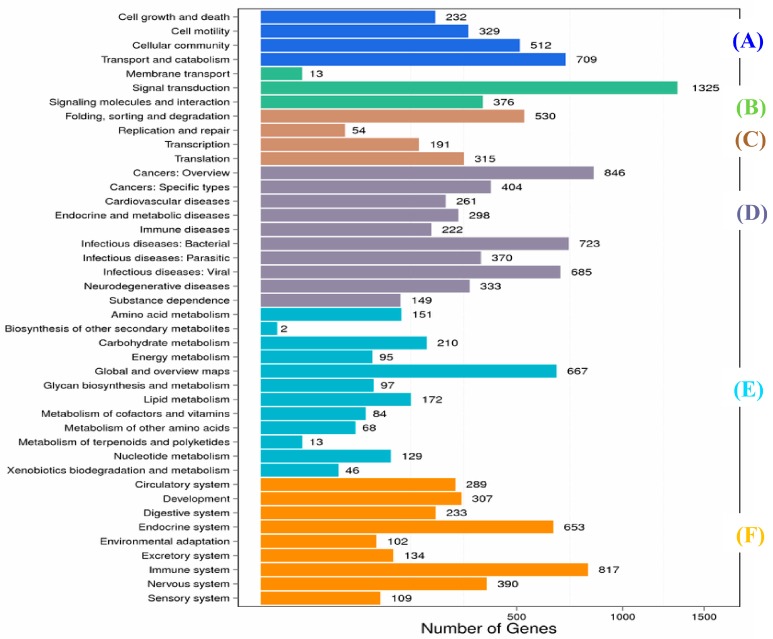
KEGG (Kyoto Encyclopedia of Genes and Genomes) classifications of DEGs (differentially expressed genes) in muscle. (**A**) Cellular processes; (**B**) environmental information processing organismal systems; (**C**) genetic information processing metabolism; (**D**) human diseases; (**E**) metabolism and (**F**) organismal systems.

**Figure 3:**
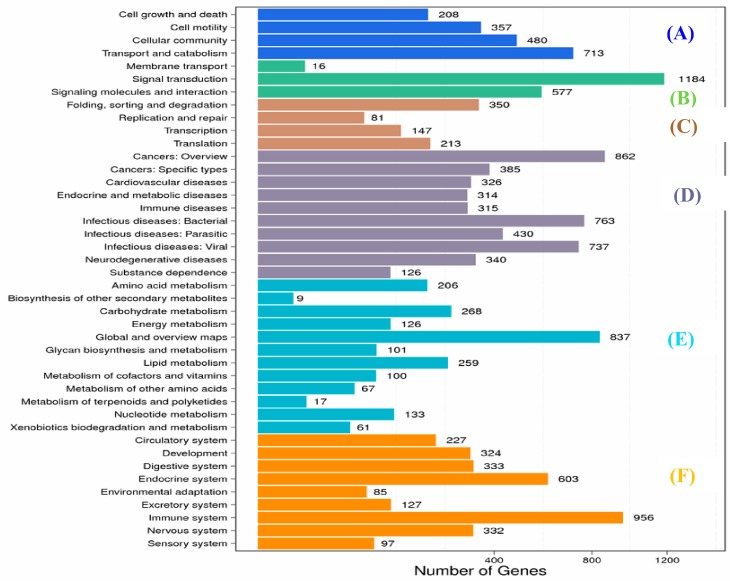
KEGG (Kyoto Encyclopedia of Genes and Genomes) classifications of DEGs (differentially expressed genes) in spleen. (**A**) Cellular processes; (**B**) environmental information processing organismal systems; (**C**) genetic information processing metabolism; (**D**) human diseases; (**E**) metabolism and (**F**) organismal systems.

Comparison of gene expression levels between the fish, subjected to bacterial challenge and control fish, identified a total of 7749 differentially expressed genes (DEGs) in the muscle and 7846 in the spleen (*p* < 0.05). This included 6300 up-regulated and 1449 down-regulated genes in the muscle, and 5111 up-regulated and 2735 down-regulated genes in the spleen, revealing a total of 7192 and 7280 (Figure 1). The DEGs in the muscle and spleen were mainly annotated into the following categories: ‘biological process’, ‘cellular component’, and ‘molecular function’ (Figure 2 and Figure 3). The most annotated unigenes belonged to the following categories: cellular process, single-organism process and metabolic process (from the ‘biological process’ category); cell, cell part and organelle (from the ‘cellular component’ category); and binding, catalytic activity and molecular transducer activity (from the ‘molecular function’ category).

Overall, from DEGs in muscle and spleen, KEGG analysis annotated the genes into 286 pathways, which were classified into 6 main categories (Figure 2 and Figure 3), namely cellular processes, environmental information processing organismal systems, genetic information processing metabolism, human diseases, metabolism, and organismal systems.

A total of 1325 DEGs in the muscle were annotated into signal transduction pathway terms with 26 sub-pathways (Supplementary Materials, Table S2a), including the PI3K Akt signaling pathway (317 genes), Rap1 signaling pathway (242 genes), MAPK signaling pathway (218 genes), Ras signaling pathway (199 genes), TNF signaling pathway (169 genes), cGMP-PKG signaling pathway (172 genes), JAK-Stat signaling pathway (142 genes), and cAMP signaling pathway (154 genes).

A total of 1184 DEGs in the spleen were annotated into 26 sub-pathways (Supplementary Materials, Table S2b), including the Rap1 signaling pathway (327 genes), PI3K Akt signaling pathway (326 genes), MAPK signaling pathway (174 genes), Ras signaling pathway (170 genes), TNF signaling pathway (145 genes), Phospholipase D signaling pathway (146 genes), Hippo signaling pathway (145 genes), phosphatidylinositol signaling pathway (146 genes), cAMP signaling pathway (136 genes), and NF-κ B signaling pathway (132 genes).

**Figure 4:**
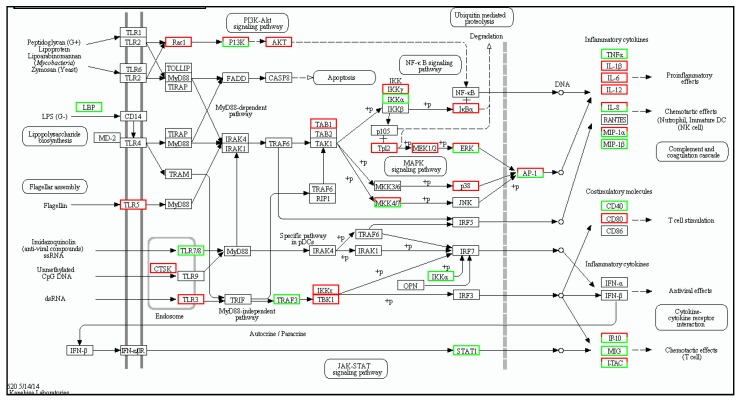
Toll-like receptor signaling mapping in spleen by KEGG. Red boxes indicate significantly differentially up-regulated expression and white boxes indicate unchanged expression in the transcriptomic profile.

**Figure 5:**
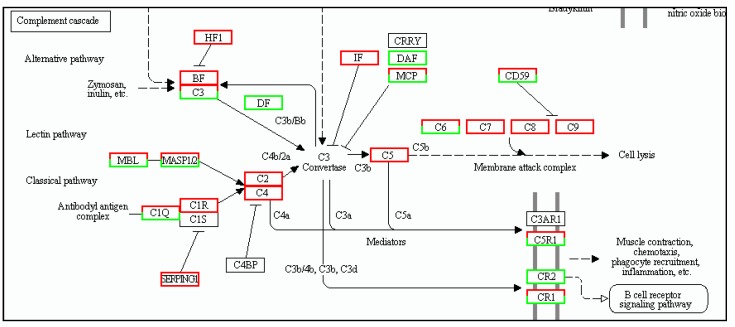
Complement cascade pathway mapping in spleen by KEGG. Red boxes indicate significantly differentially up-regulated expression, green box indicate down-regulated expression and white boxes indicate unchanged expression in the transcriptomic profile.

The DEGs of the immune system were annotated in 16 sub-categories in the spleen (S T3b), including leukocyte transendothelial migration (267 genes), platelet activation (239 genes), hematopoietic cell lineage (205 genes), T cell receptor signaling pathway (143 genes), natural killer cell-mediated cytotoxicity (171 genes), NOD-like receptor signaling pathway (80 genes), chemokine signaling pathway (164 genes), intestinal immune network for IgA production (86 genes), Fc gamma R-mediated phagocytosis (107 genes), B cell receptor signaling pathway (87 genes), antigen processing and presentation (99 genes), Toll-like receptor signaling pathway (Figure 4) (85 genes), Fc epsilon RI signaling pathway (56 genes), cytosolic DNA-sensing pathway (30 genes) and RIG-I-like receptor signaling pathway (43 genes), complement and coagulation cascades (Figure 5) (163 genes).

**Figure 6:**
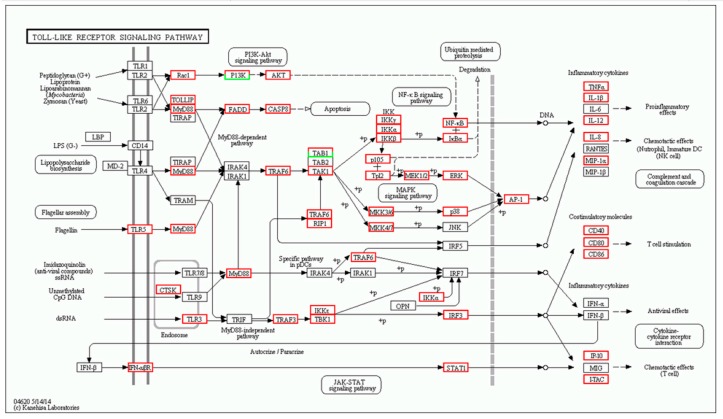
Toll-like receptor signaling mapping in muscle by KEGG. Red boxes indicate significantly differentially up-regulated expression and white boxes indicate unchanged expression in the transcriptomic profile.

**Figure 7:**
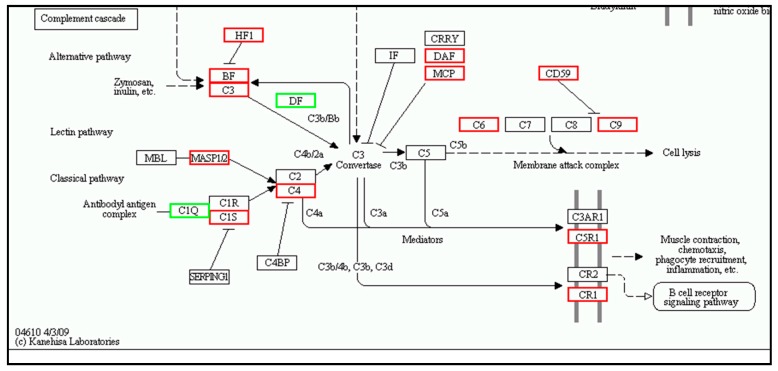
Complement cascade pathway mapping in muscle by KEGG. Red boxes indicate significantly differentially up-regulated expression, green box indicate down-regulated expression and white boxes indicate unchanged expression in the transcriptomic profile.

The immune system showed the highest number of DEGs in the muscle (817 genes); these were divided into 16 sub-categories (Supplementary Materials, Table S3a,b), including leukocyte transendothelial migration (203 genes), platelet activation (184 genes), hematopoietic cell lineage (127 genes), T cell receptor signaling pathway (147 genes), natural killer cell-mediated cytotoxicity (119 genes), NOD-like receptor signaling pathway (98 genes), Fc gamma R-mediated phagocytosis (145 genes), B cell receptor signaling pathway (107 genes), intestinal immune network for IgA production (55 genes), antigen processing and presentation (75 genes), Toll-like receptor signaling pathway (Figure 6) (114 genes), complement and coagulation cascades (Figure 7) (43 genes), Fc epsilon RI signaling pathway (76 genes), cytosolic DNA-sensing pathway (36 genes), and RIG-I-like receptor signaling pathway (65 genes).

DEGs with an absolute value of fold change >1 were selected from the immune-related category from the muscle and spleen and are presented in Table 1. Although most of the selected genes could be found in other categories, the genes were related to the complement system, antigen processing and presentation, and the Toll-like receptor signaling pathway.

### 2.3. Simple Sequence Repeat and Single Nucleotide Polymorphism Discovery

Among 47,881 unigene, 11,534 SSRs (Simple sequence repeat) were identified. The most abundant type of repeat motif was dinucleotide (44.39%), followed by trinucleotide (21.56%), quadra-nucleotide (2.05%), penta-nucleotide (0.6%), and hexa-nucleotide (0.6%) repeats (Table 2). The highest tandem repeat number was six, constituting 17.26% (Table 3).

**Figure 8:**
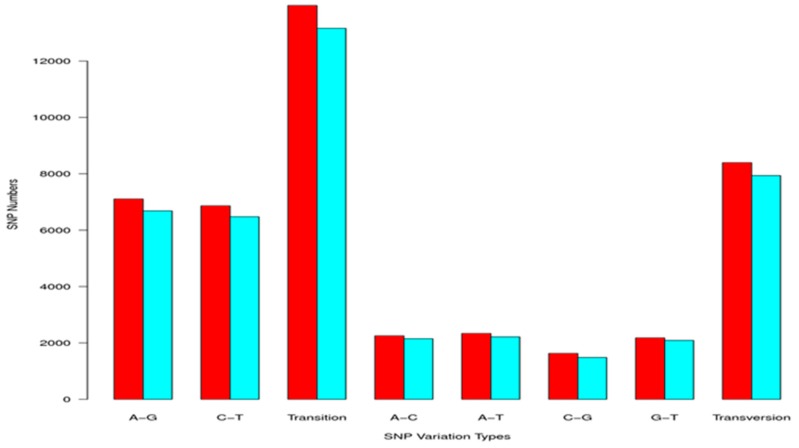
Statistics of Single nucleotide polymorphism (SNP) type in cDNA library from control and bacterial infection.

A total of 21,098 candidate SNPs were identified, transitions (13,161, 62.38%) were the most common type, and SNPs were typically located at the first and third codon positions (Figure 8).

### 2.4. RT-qPCR Analysis of Immune Related Genes Following Aeromonas sorbia Infection

To validate the DEGs identified by RNA-Seq analysis, we performed RT-qPCR. Seven genes (*C3*, *IL1β*, *IL8*, *MyD88*, *NF-κB*, *TLR5*, *TNFα*) associated with the complement system, antigen processing, and toll-like receptors were detected in the spleen and muscle by RT-qPCR (Table 4). In the spleen of the infected fish, at 1 dpi, the expression levels of *IL1β*, *IL8*, and *MyD88* were significantly up-regulated. In the muscle at 1 dpi, the expression levels of *C3*, *IL1β*, and *IL-8* showed an upward trend, but these was not significant.

## 3. Discussion

This study used the spleen and muscle of koi carp at 24 h after infection with *A. sobria* as experimental samples to determine immune-related genes and signaling pathways activated during the early stage of infection. As expected, the results showed that many immune-related genes in the koi carp were up-regulated significantly after *A. sobria* infection. The most significantly up-regulated genes associated with immunity were the pro-inflammatory cytokine-related and the signal transduction related genes, such as *IL-1β*, *TNF receptor*, *CXC chemokine*, *TGF-β*, *NF-κB*, and some other immune-related genes; pathogen recognition related genes were also significantly up-regulated.

After assembly, 69,480 unigenes were generated, with an average length of 578 bp, which was longer than those achieved in previous studies, using the Roche GS FLX 454 system with MIRA assembler (a length range of 118–2065 bp and an average length of 495 bp) [32] or Illumina/Hiseq-2000 with the assembling program-SOAP (a length range of 200–5245 bp and an average length of 412 bp) [33]. This difference in sequence quality is possibly owing to the difference in sampling tissue and the different de novo assemblers. Trinity assemblies, which were used in this study, have a consistently better performance than the other tools used in transcriptome assembling, even in the absence of a reference genome [34,35]. In contrast to Trinity, the SOAP or MIRA assemblies adopted in previous reports [32,33] were more fragmented under high values of sequencing errors and polymorphism levels [34]. Additionally, compared with the merged 69,480 unigenes in this transcriptome, previous studies provided relatively smaller gene sets (29,682 unigenes from Roche 454 system and 2139 unigenes from a SMART cDNA library) [36], and this result further emphasizes that Illumina/Hiseq-2000 RNA-Seq is a more ideal method for transcriptome analysis and it has high efficiency and massive data output.

In this study, only 60,593 unigenes were annotated with at least one database and the same problem has also been reported for the transcriptomes of other groups of marine organisms [37]. The possible reason for less annotation may be due to the availability of limited genome sequence database and research on aquaculture fish species [38,39,40]. Nonetheless, the functional annotations of unigenes according to GO, COG, and KEGG databases provided ample numbers of candidate genes and valuable information about biological features of koi carp challenged with *A. sobria* in this study. For example, as per the KEGG analysis, 33,057 sequences were assigned to 244 KEGG pathways, and among them, genetic information processing accounted for the largest number of pathways related to pathogen infection (Figure 2 and Figure 3).

In the present study, identification of SSR might be useful in genetic, evolutionary, and breeding studies [41] in koi carp studies. These data on SRR will be useful in future studies on gene expression, which can be manipulated by SRR variations in the 5 UTR regions as they affect the transcription and translation. As it is evident that SRR are ubiquitous in transcriptomes and specific, this unigene could be used for molecular marker development, comparative genetic mapping and genotyping.

## 4. Materials and Methods

### 4.1. Animal Maintenance

Prior to the conduct of experiments, all the fish were kept in a recirculatory system for 2 weeks and allowed them to acclimatize to the laboratory conditions. Throughout the experiment, fish were handled with 2-phenoxyethanol as an anesthetic. Approval for animal studies was obtained from the Center for Research Animal Care and Use Committee of the National Pingtung University of Science and Technology under protocol no #101-027 dated 19 March 2012.

### 4.2. Isolation, Cultivation, and Challenge with Aeromonas Sobria

The bacterium *A. sobria* was isolated from diseased koi carp with severe skin ulcer. The species was identified by API 20NE and 16S rDNA sequencing, cultured in brain heart infusion (BHI) broth for 18 h at 25 °C and enumerated prior to the challenge test.

A total of 15 fish (body weight 325 ± 23 g) were anaesthetized and used for intraperitoneal injection with 1 × 10^7^ cfu per fish. Individual fish received 1 × 10^7^ cfu in 200 μL phosphate buffered saline (PBS, pH 7.2). The other tank with 15 fish was injected with 200 µL of PBS (pH 7.2) only and used as a control. Three fish each from the challenge (treatment) and control groups (*n* = 3), respectively, were examined at post 24 h infection. Muscle and spleen tissues were dissected hygienically and total RNA was isolated.

### 4.3. Total RNA Isolation

TRIzol^®^ reagent (Invitrogen Corp., Carlsbad, CA, USA) was used to isolate total RNA from the tissues according to the manufacturer’s instructions. RNA integrity was assessed using the RNA Nano 6000 Assay Kit on the Bioanalyzer 2100 system (Agilent Technologies, Santa Clara, CA, USA).

### 4.4. cDNA Library Preparation and Sequencing

Genomics Bioscience Technology Co. Ltd. (Taipei, Taiwan) synthesized cDNA using 40 μg total RNA along with poly-T oligo-attached magnetic beads. First- and second-strand cDNA was synthesized using random oligonucleotides and SuperScript II reverse transcriptase. Illumina HiSeq™ 2000 (Illumina, Inc., San Diego, CA, USA) platform was used to sequence the RNA-Seq library as paired-end reads to 100 bp.

### 4.5. Assembly of De Novo Transcriptome

Adaptors and unknown bases (N) and low-quality reads were filtered using internal software and stored in FASTQ format [42]. Using the default parameter in Trinity (https://github.com/trinityrnaseq/trinityrnaseq/wiki) assembly was performed for paired reads and clustered to unigenes via *TIGR gene* indices.

### 4.6. Functional Unigene Annotation and Classification

NCBI Nr (http://www.ncbi.nlm.nih.gov/), the COG (http://www.ncbi.nlm.nih.gov/COG/), and the KEGG (http://www.genome.jp/kegg/), BlastP (Version 2.2.25) for unigenes containing open reading frames (ORFs), and BlastX for unigenes without an ORF [43] were used for annotation. The Blast2GO program was used to obtain GO annotation of the unigenes based on BLASTx hits against the NCBI Nr database [44] and aligned, and the non-aligned unigenes were predicted by ESTscan [45].

### 4.7. Identification of SSRs and SNP

SSRs were identified using MISA software package [46] (http://pgrc.ipk-gatersleben.de/misa) and di-, tri-, tetra-, penta-, and hexa-nucleotide motifs with a minimum of 8, 5, 5, 5, and 5 repeats were also identified with default parameters, respectively. SNPs were identified in the unigene of koi carp using HISAT [47] (http://ccb.jhu.edu/software/hisat/index.shtml), and then were called using GATK [48].

### 4.8. Differentially Expressed Genes and Enrichment Analysis

Bowtie2 software (http://bowtie-bio.sourceforge.net) [49] was used to determine the expression form treatment and control library. In order to obtain the DEGs in muscle and spleen tissues between the control and the infected groups, fragments per kilobase of transcripts per million fragments mapped (FPKM) values were analyzed further using the RESM [50] and the false discovery rate (FDR) was used when it is <0.05.

### 4.9. Real-Time Reverse Transcription Polymerase Chain Reaction

Once the infected fish were prepared, we performed another set of experiments for the validation of RNA-Seq data. DNase I-treated total RNA from the spleen and muscle was subjected to cDNA synthesis using iScript™ cDNA synthesis kits (Bio-Rad, Hercules, CA, USA). Reverse transcriptase real-time PCR (RT-qPCR) was performed using iQ™ SYBR^®^ Green Supermix (Bio-Rad). The list of primer sequences is shown in Table 5. Gene expression levels were normalized to that of *EF1α*. To enable comparisons between the two groups, statistical analysis was performed using Student’s *t*-test. Values of *p* < 0.05 were considered statistically significant.

## 5. Conclusions

This is the first study to provide information on host defense gene activities based on differential transcriptomic profiling in koi carp against *A. sobria*. The large number of differential expression of immune-related genes from innate immunity, antigen processing and complement cascade in this study indicated the activation of koi carp host immune response towards *A. sorbria* infection and its effect. However, further studies should be directed towards understanding of these different expressed immune genes as potential functional markers in koi carp against *A. sobria* infection. Understanding the protein levels of these functional markers in the infected koi carp would be of vital importance, as such approach may lead to a profound understanding of the regulation and responses that occur during the infection, and thus lead to the development of very specific and efficient novel vaccines and chemotherapies.

## Figures and Tables

**Table 1:** Immune-related DEGs regulated after infection in muscle and spleen of koi carp.

Gene	log2 Fold Change (Treat_muscle/PBS_muscle)	Up/Down-Regulation (Treat_muscle/PBS_muscle)	Gene	log2 Fold Change (Treat_spleen/PBS_spleen)	Up/Down-Regulation (Treat_spleen/PBS_spleen)
Innate Immunity			Innate Immunity		
*tlr5*	4.360256	Up	*tlr5*	3.787257	Up
*myd88*	2.619664	Up	*myd88*	3.701566	Up
*traf6*	1.726865	Up	*tlr7*	−3.67177	Down
*tak1*	4.95002	Up	*tbk1*	1.701046	Up
*tbk1*	2.392317	Up	*tab1*	3.308268	Up
*nfkb*	2.415037	Up	*stat1*	−1.15676	Down
*tnf-α*	9.337622	Up	*tnf*	−9.17742	Down
*il-1β*	11.21396	Up	*il-1β*	6.588696	Up
*il-12*	2.374396	Up	*il-12*	2.670009	Up
*il-8*	13.58918	Up	*il-8*	12.19013	Up
*cd40*	8.438792	Up	*cd40*	−1.38904	Down
Antigen Processing			Antigen Processing		
*tcr*	2.235073	Up	*tcr*	−4.78398	Down
*mhc-i*	7.857981	Up	*mhc-i*	11.54978	Up
*mhc-ii*	3.933396	Up	*mhc-ii*	1.144123	Up
*cd8*	7.857981	Up	*cd8*	11.54978	Up
*cd4*	3.933396	Up	*cd4*	1.144123	Up
*hsp70*	4.422052	Up	*hsp70*	2.483444	Up
Complement Cascade			Complement Cascade		
*c3*	6.954196	Up	*c3*	6.722808	Up
*c4*	1.81526	Up	*c4*	2.656724	Up
mannan-binding lectin serine protease 1	1.599362	Up	mannan-binding lectin serine protease 1	4.143954	Up
*c6*	4.712957	Up	*c6*	1.467602	Up
alpha-2-macroglobulin	5.612605	Up	alpha-2-macroglobulin	11.84314	Up

**Table 2:** Simple sequence repeat (SSR) marker discovery.

Parameter	Number
Total number of sequences examined:	47,881
Total size of examined sequences (bp):	49,734,288
Total number of identified SSRs:	11,534
Number of SSRs containing sequences:	8638
Number of sequences containing more than 1 SSR:	2103
Number of SSRs present in compound formation:	943

**Table 3:** Number of Single nucleotide polymorphism detection with the repeat number.

Repeat Number	Motif Length						Total	%
	Mono-	Di-	Tri-	Quad-	Penta-	Hexa-		
4	0	0	0	0	58	52	110	0.9
5	0	0	1214	112	5	5	1336	11.58
6	0	1358	542	78	5	8	1991	17.26
7	0	705	324	14	1	2	1046	9.06
8	0	496	242	13	0	0	751	6.51
9	0	345	31	7	0	1	384	3.32
10	0	292	34	3	0	2	331	2.86
11	0	373	33	1	0	0	407	3.52
12	802	279	27	1	1	0	1110	9.62
13	575	69	15	2	0	0	661	5.73
14	385	87	6	0	0	0	478	4.14
15	239	104	6	2	0	0	351	3.04
>15	1787	1117	19	6	0	0	2929	25.39
Total	3549	5121	2487	237	70	70	11,534	100
%	30.76	44.39	21.56	2.05	0.6	0.6	99.96	

**Table 4:** Validation of relative expression levels of immune-related genes after *A. sorbia* infection from RNA-Seq and real-time polymerase chain reaction.

Gene	Spleen		Muscle	
	RNA-Seq	qPCR	RNA-Seq	qPCR
*C3*	6.722808	2.99 ± 1.90	6.954196	2.68 ± 1.84
*IL-1β*	6.588696	* 24.73 ± 5.75	11.21396	1.23 ± 0.45
*IL-8*	12.19013	* 140.77 ± 77.4	13.58918	5.79 ± 3.82
*MyD88*	3.701566	2.92 ± 0.85	2.619664	0.76 ± 0.24
*NF-κb*	−1.15676	1.50 ± 0.58	2.415037	0.56 ± 0.16
*TLR5*	3.787257	9.14 ± 4.53	4.360256	1.08 ± 0.69
*TNFα*	−9.17742	5.33 ± 2.89	9.337622	0.25 ± 0.03

* Key gene molecules showing difference in expression level between RNA-seq and qPCR in spleen.

**Table 5:** Primer name and sequence used in the present study.

Gene		Sequence (5′-3′)
*EF1α*	Forward	5′-CCGTTGAGATGCACCATGAGT-3′
	Reverse	5′-TTGACAGACACGTTCTTCACGTT-3′
*IL-1β*	Forward	5′-GTAACGTGTGCCGGTTTCTT-3′
	Reverse	5′-GCAACACAAAAGGAAGCACA-3′
*TNFα*	Forward	5′-GCTTGTAGCTGCCGTAGGAC-3′
	Reverse	5′-GGTGGCTTGGAATTAGTG-3′
*TLR5*	Forward	5′-ATACACTCCGCTGCTGCTTT-3′
	Reverse	5′-CAAGCTGAAGGTTTCCAAGC-3′
*IL-8*	Forward	5′-GATGCAAATGCCCTCAAATACA-3′
	Reverse	5′-GGCTCTTGACGTTCCTTTTG-3′
*NF-κb*	Forward	5′-TGGCTGGAGAGGATCCATAC-3′
	Reverse	5′-AAAGCCCCTCTGTTTTGGTTG-3′
*MyD88*	Forward	5′-CAGTTCTGTGTTGCGACGTT-3′
	Reverse	5′-CGGTAAGAACTTGGCACGAT-3′
*C3*	Forward	5′-GGCTGGTCTTAGGCAGACAG-3′
	Reverse	5′-CAGCATAGGACCCGTCACTT-3′

## Supplementary Materials

Supplementary materials can be found at http://www.mdpi.com/1422-0067/19/7/2107/s1.
