# Supplementary material for: Immune-Related Functional Differential Gene Expression in Koi Carp (Cyprinus carpio) after Challenge with Aeromonas sobria
Source: Int J Mol Sci. 2018 Jul 20;19(7):2107. doi: 10.3390/ijms19072107 (PMC6073842; doi:10.3390/ijms19072107)
Supplement: Supplementary file 1 [file ijms-19-02107-s001.pdf]

Supplementary Table 1a. Length distribution of assembled transcripts and unigenes.

Sample	PBS_muscle	PBS_spleen	Treat_muscle	Treat_spleen
Total Number	39028	86458	54494	81416
Total Length	21095228	49831390	31488871	50555637
Mean Length	540	576	577	620
GC(%)	45.82	45.77	45.9	45.82

Supplementary Table 1b. Summary of annotations of assembled unigenes.

Values	Number	Percentage
Total	69,480	100%
Nr-Annotated	42,229	60.78%
Nt-Annotated	59,255	85.28%
Swissprot-Annotated	35,900	51.67%
KEGG-Annotated	33,057	47.58%
COG-Annotated	11,772	16.94%
Interpro-Annotated	32,085	46.18%
GO-Annotated	18,764	27.01%
Overall	60,593	87.21%

Supplementary table 2a. Enrichment of differentially expressed signal transduction pathway annotation in Muscle

Pathway	DEGs genes with pathway annotation (6061)	Pvalue	Qvalue
PI3K-Akt signaling pathway	317 (5.23%)	0.073479	1.59E-01
Rap1 signaling pathway	242 (3.99%)	0.949632	1.00E+00
MAPK signaling pathway	218 (3.6%)	2.31E-05	1.41E-04
Ras signaling pathway	199 (3.28%)	9.90E-05	4.98E-04
cGMP-PKG signaling pathway	172 (2.84%)	1.79E-05	1.18E-04
TNF signaling pathway	169 (2.79%)	5.14E-12	1.41E-10
Phospholipase D signaling pathway	158 (2.61%)	0.000963	3.68E-03
cAMP signaling pathway	154 (2.54%)	0.000466	1.93E-03
Jak-STAT signaling pathway	142 (2.34%)	5.15E-11	9.72E-10

Supplementary table 2b. Enrichment of differentially expressed signal transduction pathway annotation in spleen

Pathway	DEGs	Pvalue	Qvalue
Rap1 signaling pathway	327	1.98E-07	1.25E-06
PI3K-Akt signaling pathway	326	0.001518	3.99E-03
MAPK signaling pathway	174	0.136221	2.13E-01
Ras signaling pathway	170	0.029429	5.81E-02
Phospholipase D signaling pathway	146	0.005209	1.21E-02
TNF signaling pathway	145	3.26E-07	1.97E-06
Hippo signaling pathway	145	0.004469	1.05E-02
cAMP signaling pathway	136	0.016006	3.40E-02
NF-kappa B signaling pathway	132	1.11E-05	5.16E-05

Supplementary Table 3a. Analysis of differentially expressed immune relevant pathway annotation in muscle

Pathway	DEGs	Pvalue	Qvalue
Leukocyte transendothelial migration	203	0.3303246	5.57E-01
Platelet activation	184	0.862486	1.00E+00
Chemokine signaling pathway	181	2.07E-11	4.46E-10
T cell receptor signaling pathway	147	1.01E-06	8.22E-06
Fc gamma R-mediated phagocytosis	145	8.41E-12	2.12E-10
Hematopoietic cell lineage	127	0.4165758	6.42E-01
Natural killer cell mediated cytotoxicity	119	0.167073	3.28E-01
Toll-like receptor signaling pathway	114	3.14E-10	4.74E-09
B cell receptor signaling pathway	107	2.13E-08	2.39E-07
NOD-like receptor signaling pathway	98	0.108019	2.28E-01
Fc epsilon RI signaling pathway	76	8.79E-07	7.37E-06
Antigen processing and presentation	75	2.65E-08	2.86E-07
RIG-I-like receptor signaling pathway	65	0.00074621	2.93E-03
Intestinal immune network for IgA production	55	0.07092996	1.54E-01
Complement and coagulation cascades	43	0.995954	1.00E+00
Cytosolic DNA-sensing pathway	36	0.3221876	5.47E-01

Supplementary Table 3b. Analysis of differentially expressed immune relevant pathway annotation in spleen

Pathway	DEGs	Pvalue	Qvalue
Leukocyte transendothelial migration	267	4.24E-10	4.13E-09
Platelet activation	239	3.03E-05	1.31E-04
Hematopoietic cell lineage	205	1.77E-16	4.46E-15
Natural killer cell mediated cytotoxicity	171	7.72E-12	1.17E-10
Chemokine signaling pathway	164	1.21E-08	9.60E-08
Complement and coagulation cascades	163	9.08E-41	2.74E-38
T cell receptor signaling pathway	143	4.75E-07	2.76E-06
Fc gamma R-mediated phagocytosis	107	0.001148	3.21E-03
Antigen processing and presentation	99	5.15E-21	2.59E-19
B cell receptor signaling pathway	87	0.00033	1.05E-03
Intestinal immune network for IgA production	86	5.21E-11	6.55E-10
Toll-like receptor signaling pathway	85	0.001296	3.55E-03
NOD-like receptor signaling pathway	80	0.662791	7.47E-01
Fc epsilon RI signaling pathway	56	0.017228	3.61E-02
RIG-I-like receptor signaling pathway	43	0.491475	5.96E-01
Cytosolic DNA-sensing pathway	30	0.656418	7.42E-01

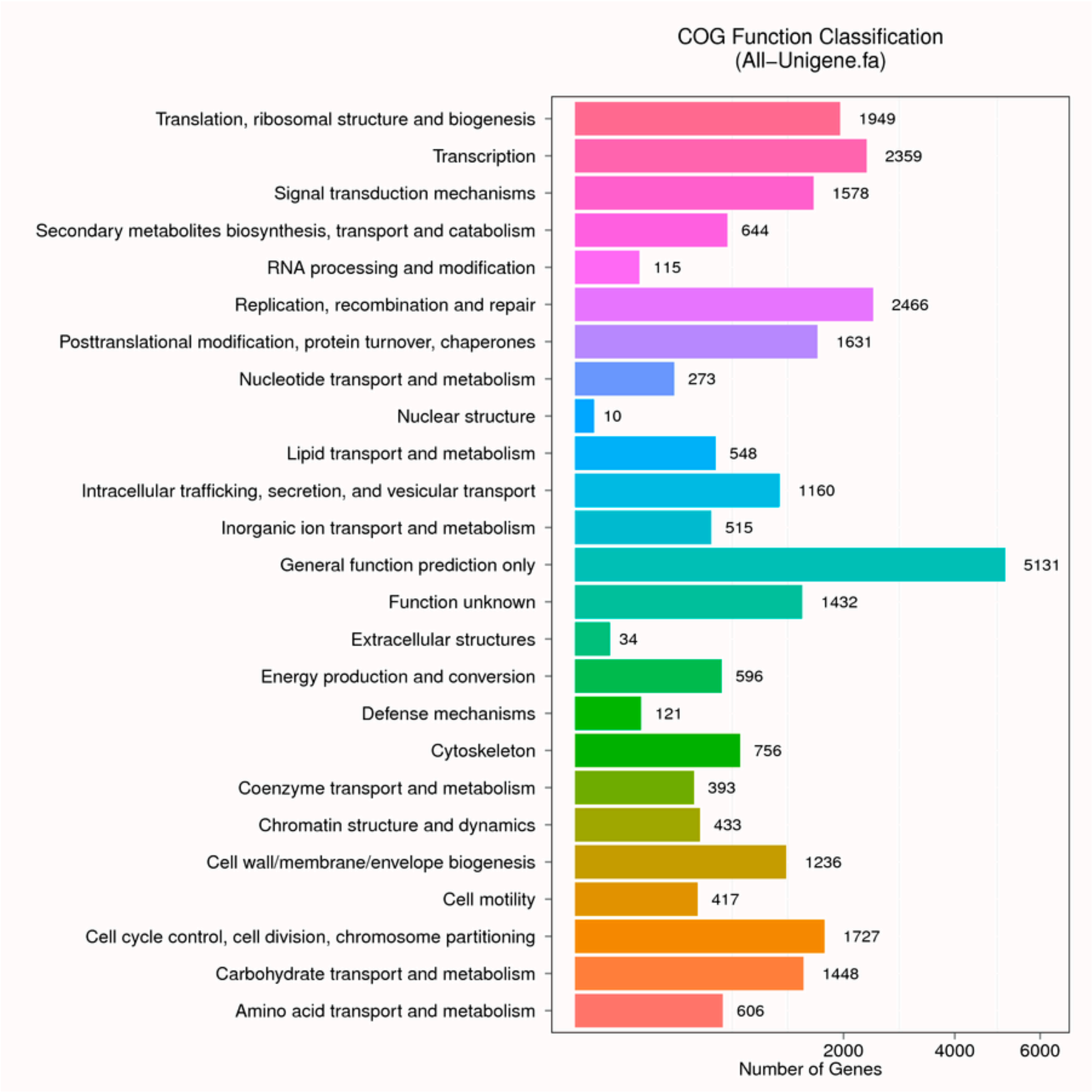

Supplementary figure 1. The cluster of orthologous groups (COG) classification.
